# Effects of extreme temperatures on public sentiment in 49 Chinese cities

**DOI:** 10.1038/s41598-024-60804-1

**Published:** 2024-04-30

**Authors:** Chan Wang, Yi-Xiang Bai, Xin-Wu Li, Lu-tong Lin

**Affiliations:** 1 School of Economics, Guangdong University of Finance and Economics, Guangzhou, 510320 People’s Republic of China; 2 School of Economics, Nankai University, Tianjin, 300071 People’s Republic of China

**Keywords:** Climate-change impacts, Climate-change mitigation, Psychology and behaviour

## Abstract

The rising sentiment challenges of the metropolitan residents may be attributed to the extreme temperatures. However, nationwide real-time empirical studies that examine this claim are rare. In this research, we construct a daily extreme temperature index and sentiment metric using geotagged posts on one of China's largest social media sites, Weibo, to verify this hypothesis. We find that extreme temperatures causally decrease individuals' sentiment, and extremely low temperature may decrease more than extremely high temperature. Heterogeneity analyses reveal that individuals living in high levels of PM2.5, existing new COVID-19 diagnoses and low-disposable income cities on workdays are more vulnerable to the impact of extreme temperatures on sentiment. More importantly, the results also demonstrate that the adverse effects of extremely low temperatures on sentiment are more minor for people living in northern cities with breezes. Finally, we estimate that with a one-standard increase of extremely high (low) temperature, the sentiment decreases by approximately 0.161 (0.272) units. Employing social media to monitor public sentiment can assist policymakers in developing data-driven and evidence-based policies to alleviate the adverse impacts of extreme temperatures.

## Introduction

Climate change significantly impacts the economy, agriculture, and residents' daily life^1–6^. In recent years, the frequent catastrophic weather events have significantly affected biodiversity^7^, countries' economic development, and residents' health^8–10^. With greenhouse gas emissions at record levels^11,12^, there has been a discernible rise in the occurrence of extreme temperatures' events, such as hot days and nights^13–15^, as well as freezing days^16^. Extreme temperatures exert a tremendous impact on the well-being and survival of residents. It has been revealed that extreme temperatures significantly affect physiological health^17,18^. These effects, in turn, consequently have a negative impact on mental health^19^, such as reducing residents' sentiment. It is essential to assess the effects of extreme temperatures on citizens' sentiment for formulating evidence-based policies, particularly under the background of the high frequency of extreme temperature events.

Analyzing social media data could assist policymakers in comprehending individuals' sentiment and behaviors, enhancing their comprehension of how policies impact social phenomena^20,21^. This study uses social media data to reflect public sentiment accurately. In light of the recurrent extreme temperature events and the COVID-19 pandemic, numerous studies have utilized social media data to examine how these factors impact residents' sentiment^21–24^. Moreover, various studies have employed social media data to uncover that climate change elicits more negative sentiment from individuals^25–29^. Nevertheless, there was a lack of research investigating the real-time impacts of extreme temperatures on sentiment. Hence, we aim to contribute this study to the existing research in the following areas. Firstly, this study improves the measurement of extreme temperatures compared to prior research. To quantify the severity of a day's temperature, the difference between the daily temperature threshold of each city and the actual temperature recorded on that day is utilized in this paper. Secondly, this study enhances existing research by indicating how sentiment evolved in reaction to the fluctuating extreme temperatures over time, employing robust methodologies. Thirdly, this research examines the discrepancies in the impact of extreme temperatures on the sentiment of individuals who reside in various environmental and urban settings. For instance, centralized heating and air conditioning may mitigate the adverse impact of extreme temperatures on residents' sentiment^30,31^.

## Data and method

**Figure 1:**
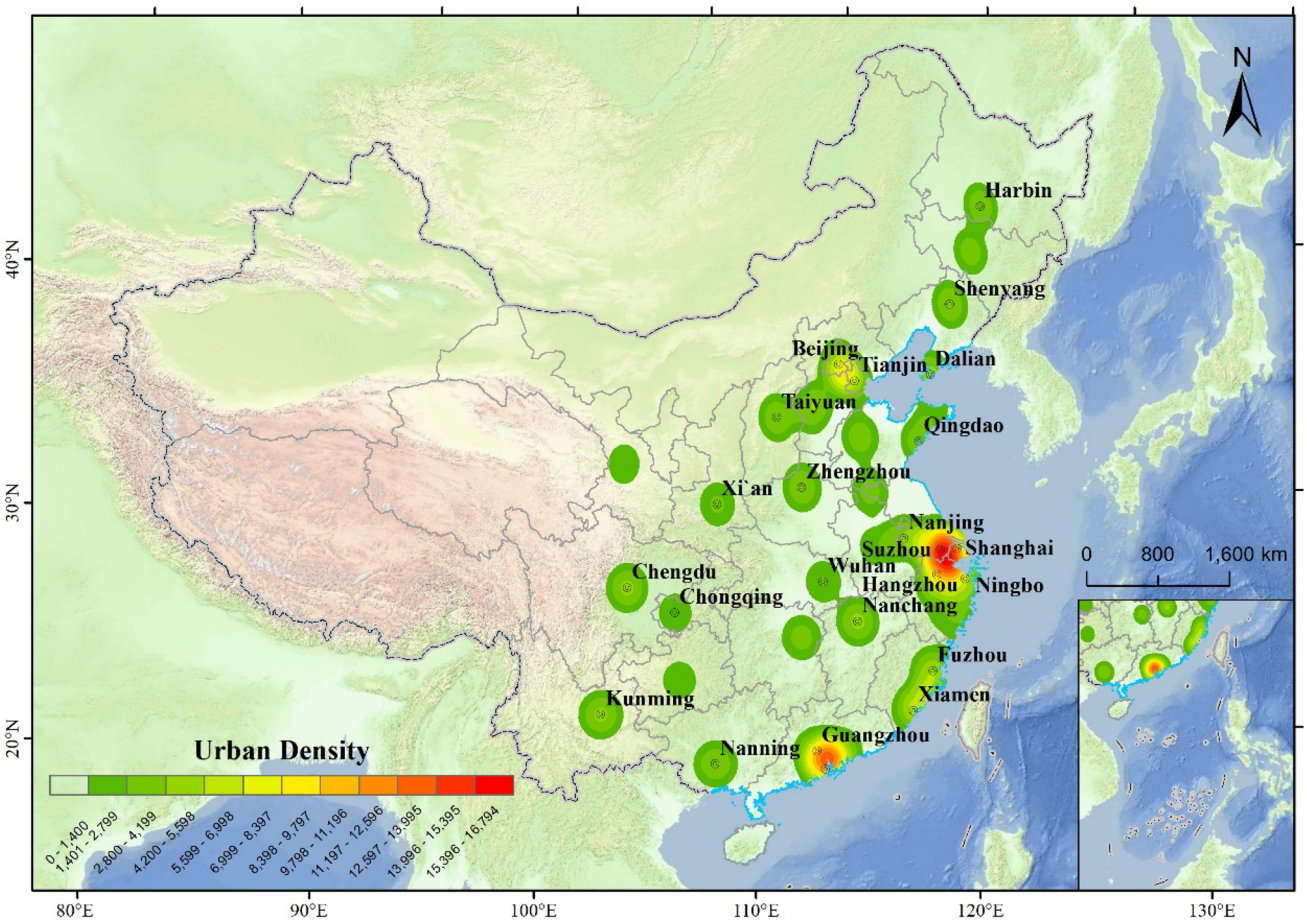
The distribution of 266,744 Weibo posts. Posts used in this paper are concentrated in three regions: the Beijing-Tianjin-Hebei urban agglomeration, the Yangtze River Delta urban agglomeration, and the Pearl River Delta urban agglomeration. Completely covering the largest urban agglomerations in China's north and south, it provides rich data for heterogeneity analysis. Image was drawn using ArcGIS Pro 3.0.2 software by authors.

This study investigated the impact of extreme temperatures on the residents' sentiment in 49 cities, employing daily data from Weibo, China's largest microblogging social media platform^32^. Studying China is quite beneficial due to its diverse climate conditions^29^. Furthermore, compared to other cities, these 49 selected cities, including Tier 1, New Tier 1, and Tier 2 cities (1A section in SI Appendix), have larger populations and higher volumes of Weibo, thus ensuring greater robustness and richness in our findings. The study examined 497,736 posts from 49 main cities in China, spanning 12 months in 2020. The posts from cities' topics were intended to capture the changes within the cities instead of other extraneous topics. Subsequently, in order to eradicate the disturbances generated by redundant advertising posts, we applied a filtering process to eliminate duplicate posts, resulting in a dataset consisting of 266,744 posts (Fig. 1). The posts were categorized according to both geographical area and date in order to gather all pertinent posts for each city on specific days throughout 2020. This study evaluated the sentiment of each post utilizing Tencent's NLP platform sentiment analysis interface, which features a machine-trained sentiment analysis algorithm^28^. In detail, the sentiment analysis interface can analyze, process, summarize, and reason about subjective text with emotional overtones, identifying the probability score that writer's emotional disposition is positive. The score indicated the sentiment value given in a Weibo post. According to existing research, the sentiment index for a city on a specific day was determined by using the median sentiment value for that city on that day^28^. Comparing the average sentiment to the middle sentiment value, the median sentiment can be more helpful in eliminating the influence of extreme sentiment values. The indicator is measured on a scale of 0 to 100, where 0 represents a profoundly negative feeling, and 100 represents a significantly positive emotion. Furthermore, the subsequent models include the sentiment index as the dependent variable.

**Figure 2:**
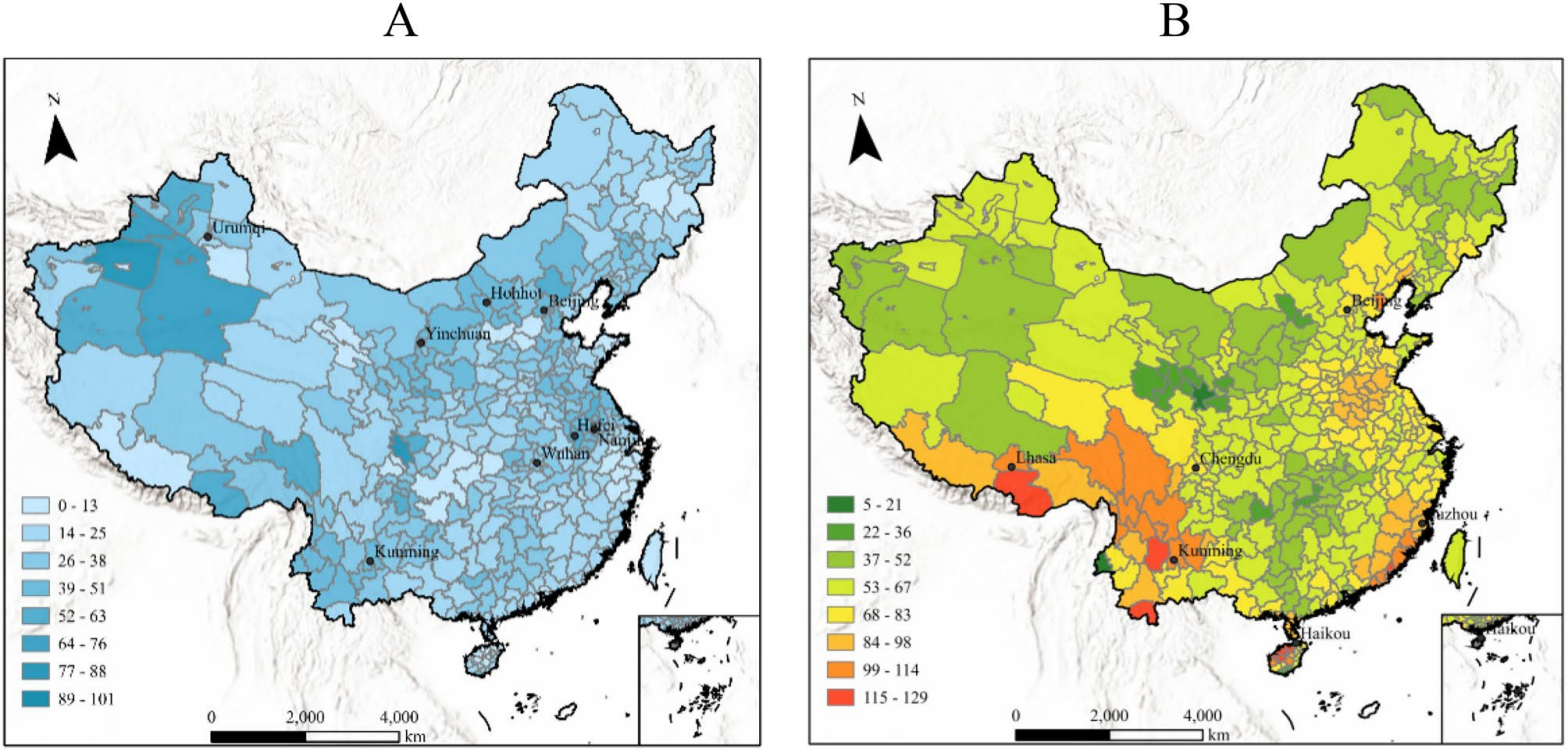
(**A**) The distribution of the CNI across China in 2020. This study utilized the cold night index of 340 cities in China to obtain the distribution of CNI across China in 2020. (**B**) The distribution of the WDI across China in 2020. This study utilized the WDI of 340 cities in China to obtain the distribution of warm day across China in 2020. Images were drawn using ArcGIS Pro 3.0.2 software by authors.

This study devised a collection of indicators to measure extreme temperatures in each city. Typically, a historical percentile threshold temperature for a given duration of time is used as the standard to determine if extreme temperature events occur. Extreme high-temperature events, also known as warm days, are characterized by temperatures beyond a historical threshold temperature, such as the 99th or 95th percentile, over a specific duration of time^33^. Similarly, when the temperature falls below a historical threshold temperature, such as the 1st or 5th percentile, it can be observed as an extreme low-temperature event, also known as a cold night^34,35^. This study utilized the 95th and 5th percentile threshold temperatures from twenty years as benchmarks. This research examined temperature data from 340 cities in China to ascertain the occurrence of warm days and cold nights in 2020. Subsequently, the Warm Day Index (WDI) and Cold Night Index (CNI) for each city in 2020 were developed. Figure 2 displays the data from 340 cities in China throughout 2020, showing the distributions of WDI (Fig. 2A) and CNI (Fig. 2B). Moreover, the linear difference method was employed to calculate the difference between the highest (lowest) temperature and the standard of extremely high (low) temperature in order to reflect the extremely high (low) temperature. The definitions of extreme temperatures are shown in formula (1) and (2).

This research employed rigorous econometric techniques to evaluate causal correlations between extreme temperatures and sentiment reported on social media. We selected control variables from multiple perspectives based on existing research to mitigate the impact of confounding factors that may affect residents' sentiment. The first set of control variables consisted of different natural environment variables, such as precipitation, wind speed, and air quality (measured by PM2.5)^28^. The second set of control variables consisted of significant public event variables, such as the total count of confirmed COVID-19 cases^36^. The correlation test results between the variables are shown in the second section in SI Appendix.

### Definition of extreme temperatures

The temperature data employed in this study are sourced from the National Oceanic and Atmospheric Administration (NOAA) of the National Environmental Information Centre (NCEI). The data for the highest and lowest temperatures has been grouped by cities. The highest temperature for a city on a given day was determined by calculating the average of the highest temperature data from all the weather stations in that city. Likewise, we acquired the cities' daily lowest temperature data using the identical approach. Then, we utilized the highest temperature subtract the standard of 95% of the city's highest temperature for the day from 2000 to 2019 to obtain the index, which shows the degree to which the daily highest temperature is above the daily extreme high-temperature standard ($95\% EHTit$). We then employed the difference between the standard of 5% of the city's minimum temperature for the day from 2000 to 2019 and the daily minimum temperature of the city, indicating the degree that the daily minimum temperature falls below the daily extreme low-temperature standard ($5\% ELTit$). Furthermore, combined with speed of wind, we calculated extremely high-apparent temperature ($EHATit$) and extremely low-apparent temperature ($ELATit$). Moreover, we extended the given duration of time to 30 years (1990–2019), and calculated another extreme temperatures index in the same way ($90\% TEHTit$, $10\% TELTit$). $95\% EHTit$, $5\% ELTit$, $EHATit$ and $ELATit$ are defined by Eqs. (1–4) separately.

1 $$95\% EHT_{it} = MAXT_{it} - Max_{95\% }$$

2 $$5\% ELT_{it} = Min_{5\% } - MINT_{it}$$

3 $$EHAT_{it} = EHT_{it} - \sqrt {WS_{it} }$$

4 $$ELAT_{it} = ELT_{it} - \sqrt {WS_{it} }$$

### Multiple linear regression (MLR)

In order to examine the impact of extreme temperatures on individuals' sentiment, we employed a fixed-effects panel regression approach to estimate Eq. (5). $Sentimentit$, $EHTit$ and $ELTit$ respectively represent sentiment index, the extremely high temperature and the extremely low temperature of city $i$ on day $t$. $Xit$ indicates weather conditions, and $Tt$ is used to control for week fixed effects, where $T$ refers a serious of weekdays indicator variables. Moreover, $\gamma_{i}$ is used as a city fixed effect to control for time-invariant unobservable factors that vary across cities.

5 $$Sentiment_{it} = \beta_{0} + \beta_{1} EHT_{it} + \beta_{2} ELT_{it} + \beta_{3} X_{it} + T_{t} + \gamma_{i} + \varepsilon_{it}$$

### Instrumental variables (IV)

Drawing on related studies^28^, we employed data from neighboring cities to simulate explanatory variables in target cities. Suppose cities bordering the target city are at the same altitude as the target city. In that case, the temperatures of these cities are comparable to the target city and can be used to synthesize the target city temperature. Additionally, as an ideal IV, temperatures in other cities are unlikely to impact the target city's local social and economic activities via other channels. Therefore, we performed synthetic simulations of the temperature of the target city using the temperature of other cities bordering the target city. The synthesized simulated extremely high temperatures and extremely low temperatures were defined as $NEHTit$ and $NELTit$. According to the theorem that the temperature decreases by 0.6 °C for every 100 m of altitude increase, we recalculated the temperature of city* j* when the simulated city *j* were at the altitude level of the target city *i*, and then eliminated the effect on the temperature caused by the different altitudes of the city.

6 $$NEHT_{it} = average\left( {\sum\limits_{j} {\left[ {EHT_{ijt} - 0.6(Al_{j} - Al_{i} )/100} \right]} } \right)$$

7 $$NELT_{it} = average\left( {\sum\limits_{j} {[ELT_{ijt} } - 0.6(Al_{j} - Al_{i} )/100]} \right)$$

where $EHT_{ijt}$ represents the extremely high temperature of city* j*, bordering city *i* on day* t*, and $ELT_{ijt}$ also represents the extremely low temperature of city *j*, bordering city *i* on day* t*.

### Definition of extreme temperature differences

Similar to the definition of extreme temperatures, the difference between the maximum temperature difference and the 95% maximum temperature difference on the selected day and city was defined as $EHTD_{it}$ in formula (8). Moreover, the difference between the 5% minimum temperature and the minimum temperature difference on the selected day and city was defined as $ELTD_{it}$ in formula (9).

8 $$EHTD_{it} = MAXTD_{it} - MaxTD_{95\% }$$

9 $$ELTD_{it} = MinTD_{5\% } - MINTD_{it}$$

## Results

MLR was utilized to determine the correlation between people's sentiment and extreme temperatures, including extremely high and low temperatures, in 49 cities in 2020. Moreover, we employed MLR to examine the impact of other extreme temperature variables on residents' sentiment, such as the extreme temperature difference between neighboring days and extremely apparent temperatures.

### Impacts of extreme temperatures on sentiment

Each column in Table 1 reports the standardized coefficient and *P*-values based on model (5). This regression analysis was controlled for city and week fixed effects while also incorporating control variables (1B section in SI Appendix). This paper employed the overall sentiment index as the dependent variable, which is determined by computing the median sentiment value for a specific city on a given day. The dataset comprises 16,944 observations from 49 cities in 2020. Initially, we utilized $95\% EHTit$ and $5\% ELTit$ as explanatory variables (2nd column of Table 1). Subsequently, robustness checks were conducted using $90\% EHTit$ and $10\% ELTit$ as explanatory variables (3th of Table 1). Considering that wind speed may affect the human body's perceived temperature, we introduced wind speed to construct extremely apparent temperature indicators including $95\% EHATit$, $5\% ELATit$, $90\% EHATit$ and $10\% ELATit$ (4th and 5th column of Table 1).

**Table 1:** The regression results of extreme temperature and extremely apparent temperature.

Sample	Sentiment	Sentiment	Sentiment	Sentiment
$95\% EHTit$	− 0.1606***			
	(0.0273)			
$5\% ELTit$	− 0.2724***			
	(0.0356)			
$90\% EHTit$		− 0.1490***		
		(0.0312)		
$10\% ELTit$		− 0.2936***		
		(0.0426)		
$95\% EHATit$			− 0.1610***	
			(0.0403)	
$5\% ELATit$			− 0.2718***	
			(0.0626)	
$90\% EHATit$				− 0.1494 ***
				(0.0444)
$10\% ELATit$				− 0.2930***
				(0.0725)
City FE	Yes	Yes	Yes	Yes
Time FE	Yes	Yes	Yes	Yes
Control Variables	Yes	Yes	Yes	Yes
N	16,944	16,944	16,944	16,944
R^2^	0.2822	0.2813	0.2822	0.2813

The robust standard error is clustered by city. Standard Errors are shown in parentheses, *P < 0.1; **P < 0.05; ***P < 0.01.

According to the results of Table 1, a negative correlation exists between sentiment and extreme temperatures ($95\% EHTit$,$5\% ELTit$,$90\% EHTit$, $10\% ELTit$). Extreme temperatures significantly reduce the sentiment score of residents. The findings indicate that for a one-standard increase in the $5\% ELTit$, there is a corresponding decrease of 0.2724 units in the sentiment score; while a one-standard unit increase in the $95\% EHTit$ corresponds to a decrease of 0.1606 units in the sentiment score. Similarly, a one-standard increase in $90\% EHTit$ is associated with a reduction of 0.1490 units in residents' sentiment score while a one-standard increase in $10\% ELTit$ leads to a decrease of 0.2936 units in residents' sentiment. Our findings indicate that when loosening the criteria for defining extremely high and low temperatures, individuals may exhibit increased sensitivity towards extremely low temperature but decreased sensitivity towards extremely high temperature.

### Robustness checks

The MLR analysis revealed the connections between sentiment and extreme temperatures. To strengthen the reliability of the findings, we employed the Baidu NLP tool to generate an additional sentiment score using the same procedures. This score was then incorporated into the regression model as an explanatory variable (5) to verify the conclusions further. Subsequently, a rigorous assessment was performed using temperature data from the Met Office. The negative correlations have been determined to remain valid (Table 2).

**Table 2:** The regression results of robustness check.

Dependent variables	Sentiment	Sentiment	Robust sentiment
$95\% EHTit$	− 0.1606***		− 0.1419***
	(0.0273)		(0.0409)
$5\% ELTit$	− 0.2724***		− 0.1331***
	(0.0356)		(0.0493)
$95\% REHTit$		− 0.1224***	
		(0.0350)	
$5\% RELTit$		− 0.2190***	
		(0.0520)	
City FE	Yes	Yes	Yes
Time FE	Yes	Yes	Yes
N	16,944	16,944	16,944
R^2^	0.2822	0.2818	0.1526

Standard Errors are shown in parentheses, *P < 0.1; **P < 0.05; ***P < 0.01.

The negative link between extreme temperatures and citizens' sentiment may be attributed to omitting varying daily factors at the city level. The urban heat island effect, a result of urbanization closely associated with economic expansion, may impact individuals' sentiment. Given this scenario, our assessments would be inclined to ascribe negative sentiment exclusively to extremely high and low temperatures, resulting in a biased estimation. In order to tackle this possible problem of endogeneity, the researchers utilized the IV technique. Despite implementing this method, extreme temperatures still negatively impacted sentiment, as indicated in Table 3. The results of the unidentifiable test and the weak IV test are shown in the third section SI Appendix.

**Table 3:** The result of regression of baseline model.

Dependent variables	Sentiment	Sentiment	Sentiment	Extremely High temperature	Extremely low temperature	Sentiment
	OLS	OLS	OLS	Stage 1 of IV	Stage 1 of IV	Stage 2 of IV
$95\% EHTit$	− 0.1606***	− 0.6586***	− 0.4429***			− 0.1861***
	(0.0273)	(0.1566)	(0.1564)			(0.0323)
$5\% ELTit$	− 0.2724***	− 0.7713***	− 0.5360***			− 0.3766***
	(0.0356)	(0.1794)	(0.1794)			(0.0391)
$NEHTit$				0.9819***	0.0005	
				(0.0024)	(0.0018)	
$NELTit$				− 0.0022	0.9837***	
				(0.0029)	(0.0022)	
$PM2.5it$	0.0153***	0.0144***	− 0.0066	− 0.0005	0.0004*	0.0160***
	(0.0038)	(0.0038)	(0.0042)	(0.0003)	(0.0002)	(0.0041)
$WSit$	0.1576***	0.4883***	0.3645***	0.0077*	− 0.0044	0.5501***
	(0.0564)	(0.1321)	(0.1327)	(0.0044)	(0.0033)	(0.0568)
$95\% EHTWSit$		0.0336**	0.0231*			
		(0.0134)	(0.0134)			
$5\% ELTWSit$		0.0311*	0.0217			
		(0.0160)	(0.0160)			
$PCDIi$		− 0.0001***	− 0.0001***			
		(0.00002)	(0.00002)			
$95\% EHTPCDIit$		0.0033**	0.0035**			
		(0.0015)	(0.0015)			
$5\% ELTPCDIit$		0.0034**	0.0040**			
		(0.0016)	(0.0016)			
$Pit$	− 0.0520***	− 0.0520***	− 0.0340***	− 0.0119***	0.0043***	− 0.0467***
	(0.0105)	(0.0110)	(0.0114)	(0.0010)	(0.0007)	(0.0128)
$CCDit$	− 0.0175**	− 0.0166**	− 0.0140**	− 0.0006	0.0010**	− 0.0730***
	(0.0071)	(0.0071)	(0.0071)	(0.0005)	(0.0004)	(0.0069)
Constant	30.7237***	54.3397***	58.8498***	0.1680	− 0.0013	40.2316***
	(0.8032)	(2.0575)	(2.0967)	(0.0352)	(0.0268)	(0.4574)
City FE	Yes	Yes	Yes	Yes	Yes	Yes
Time FE	Yes	Yes	No	Yes	Yes	Yes
Solar terms FE	No	No	Yes	No	No	No
N	16,944	16,944	16,944	16,944	16,944	16,944
R^2^	0.2822	0.2829	0.2960	0.9138	0.9306	0.0205

Standard Errors are shown in parentheses, *P < 0.1; **P < 0.05; ***P < 0.01.

To further validate the robustness of the extreme temperature indicator, the 90th and 10th historical percentile threshold temperature for 30 years were used to recalculate extremely high temperature ($90\% TEHTit$) and extremely low temperature ($10\% TELTit$)^37^. The results were shown in Table 4. It was found that the adverse effects of extreme temperatures on sentiment were still significantly present.

**Table 4:** The result of extreme temperature's robustness check.

Dependent variables	Sentiment	Sentiment	Sentiment	Robust Sentiment
	OLS	OLS	OLS	OLS
$90\% TEHTit$	− 0.1777***	− 0.6694***	− 0.4095***	− 0.0395***
	(0.0272)	(0.1553)	(0.1587)	(0.0091)
$10\% TELTit$	− 0.1456***	− 0.4985***	− 0.3410*	− 0.0316***
	(0.0327)	(0.1895)	(0.1912)	(0.0120)
$PM2.5it$	0.0180***	0.0164***	− 0.0065	0.0037***
	(0.0038)	(0.0038)	(0.0043)	(0.0012)
$WSit$	0.1745**	0.5189***	0.3581***	0.0466**
	(0.0070)	(0.1304)	(0.1325)	(0.0198)
$90\% TEHTWSit$		0.0342***	0.0190	
		(0.0130)	(0.0133)	
$10\% TELTWSit$		0.0279**	0.0209	
		(0.0129)	(0.0129)	
$Pit$	− 0.0572***	− 0.0574***	− 0.0337***	− 0.0149***
	(0.0105)	(0.0105)	(0.0107)	(0.0052)
$CCDit$	− 0.0165**	− 0.0160**	− 0.0148**	− 0.0018
	(0.0070)	(0.0070)	(0.0071)	(0.0031)
$PCDIi$		− 0.0001***	− 0.0001***	
		(0.0000)	(0.0000)	
$90\% TEHTPCDIit$		0.0032**	0.0035**	
		(0.0014)	(0.0015)	
$10\% TELTPCDIit$		0.0021	0.0027	
		(0.0017)	(0.0017)	
Constant	30.4437***	53.7734***	58.9532***	95.0950***
	(0.8347)	(2.4432)	(2.4975)	(0.3417)
City FE	Yes	Yes	Yes	Yes
Time FE	Yes	Yes	No	Yes
Solar terms FE	No	No	Yes	No
N	16,944	16,944	16,944	16,944
R^2^	0.2809	0.2816	0.2959	0.0980

Standard Errors are shown in parentheses, *P < 0.1; **P < 0.05; ***P < 0.01.

### Impacts of extreme temperature differences on sentiment

While the sentiment might be influenced by fluctuations in neighboring extreme temperatures, extreme temperature differences were introduced as explanatory variables to investigate further the relationship between residents' sentiment and extreme temperatures. $EHTDit$ and $ELTDit$ were replaced for regression. It was found that extremely high-temperature difference and extremely low-temperature difference from neighboring days played a negative role in the sentiment (Table 5). Moreover, residents appear more sensitive to $ELTDit$ (− 0.2003) than $EHTDit$(− 0.1177). Similarly, we analyzed the effects of extremely high-apparent-temperature difference and low-apparent-temperature difference. It was revealed that extremely apparent-temperature differences also have a negative impact on sentiment (Table 5). It has been suggested that global warming's effect on human thermal comfort could increase heat stress and decrease cold stress^38^.

**Table 5:** The result of regression of extended model.

Dependent variables	Median sentiment	Median sentiment
	OLS	OLS
$EHTDit$	− 0.1177***	
	(0.0351)	
$EHATDit$		− 0.0976***
		(0.0289)
$ELTDit$	− 0.2003***	
	(0.0409)	
$ELATDit$		− 0.2401***
		(0.0452)
$PM2.5it$	0.0201***	0.0200***
	(0.0037)	(0.0036)
$WSit$	0.1648***	0.0451
	(0.0576)	(0.0613)
$Pit$	− 0.0495***	− 0.0494***
	(0.0112)	(0.0112)
$CCDit$	− 0.0164**	− 0.0172**
	(0.0071)	(0.0071)
Constant	30.8834***	30.8989***
	(0.9095)	(0.9159)
City FE	Yes	Yes
Time FE	Yes	Yes
N	16,944	16,944
R^2^	0.2801	0.2803

Standard Errors are shown in parentheses, *P < 0.1; **P < 0.05; ***P < 0.01.

### Interactive effect

The degree of impacts of extreme temperatures on sentiment might be modified by several factors including wind speed, per capita disposable income and so on. Furthermore, we incorporated multiple interaction variables into the baseline model to examine the relationships to analyze. The results are shown in Table 3. The study revealed that the influence coefficients of extreme temperatures on sentiment might be affected by wind speed and per capita disposable income. In addition, the model incorporated the fixed effects of the 24 solar terms instead of the fixed effects of week. The adverse impacts of extreme temperatures on sentiment persisted.

### Heterogeneity analysis

**Figure 3:**
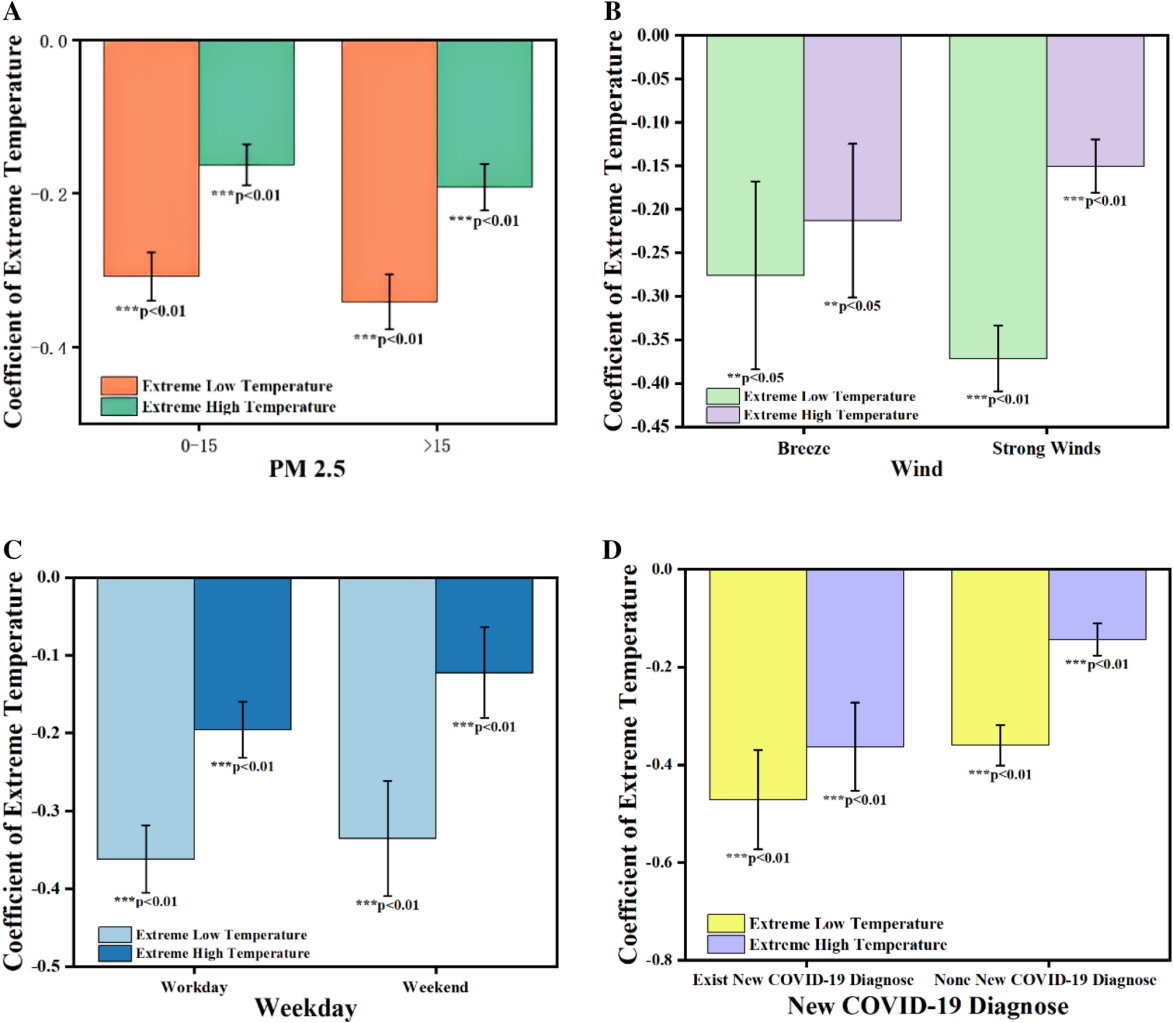
The coefficients of extreme temperatures under different conditions. (**A**) PM2.5 level. (**B**) Wind speed level. (**C**) Weekdays or Weekends. (**D**) New COVID-19 diagnose. Images were drawn using Origin 2018 software by authors.

Moreover, the residents' sentiment is also influenced by other meteorological factors, holidays, weekdays, and significant public events. To comprehensively reveal the disparities in the impact of extreme temperatures on sentiment under various conditions, heterogeneity analysis was employed for investigation in this paper. PM2.5 can detrimentally affect residents' health, leading to a decrease in their sentiment. As per the 2021 World Health Organization (WHO) Global Air Quality Guidance (AQG) published in 2021, which set a target daily average PM2.5 level at 15 μg/m^3^^39^, we divided the dataset into two groups based on the threshold value of PM2.5 (Fig. 3A). The results indicated that when PM2.5 levels exceeded 15 μg/m^3^, individuals exhibited greater sensitivity to both extremely high temperature (-0.192) and extremely low temperature (-0.341) compared to when PM2.5 levels were below 15 μg/m^3^, where the sensitivity to extremely high temperature was -0.162 and extremely low temperature was − 0.308. It was revealed that extreme temperatures have a more detrimental impact on sentiment when air quality worsens.

The variation in wind speed may lead the disparity between the ambient temperature and the subjective temperature experienced by individuals. Based on the Beaufort Wind Rating Table (BWRT), wind speeds equal to or less than 3.3 m/s are categorized as wind power level 2 and below, while wind speeds above 3.3 m/s fall under wind power level 3 and above. According to the definition of wind speed, we categorized the dataset into two groups for regression analysis using a threshold of 3.3 m/s. The control group consisted of observations with wind speeds below 3.3 m/s. The experimental group included observations with wind speeds equal to or greater than this threshold (Fig. 3B). Our findings indicated that the experimental group has a larger extreme high-temperature coefficient (− 0.150) compared to the control group (− 0.213). However, the control group has a larger extreme low-temperature coefficient (− 0.276) than the experimental group (− 0.371). Research findings indicated that the effect of extremely high temperature on residents' sentiment was less pronounced in a wind level 3 and above than in a wind level 2 and below. Nevertheless, the impact of extremely low temperatures was contrary.

On weekdays, residents have limited autonomy in their outdoor activities, whereas on weekends, they could decide whether go outside or not depending on the temperature. It was revealed that residents' sentiment was more sensitive to extreme temperatures on weekdays compared to weekends (Fig. 3C). Besides, public emergencies could exacerbate the impact of extreme temperatures on residents' sentiment. For instance, unexpected major public events such as epidemics^36,40–42^, stock market fluctuations^43^, and hosting of the Olympic Games^44^ can affect residents' sentiment. On days when there are adverse news events, the impact of extreme temperatures on residents' sentiment is further magnified. This study chose new confirmed cases of COVID-19 diagnoses as unexpected major public events. In cities where new cases of COVID-19 were reported, the coefficient of extremely high temperature (− 0.363) was lower compared to cities without new cases (− 0.143) (Fig. 3D). The results of the coefficient of extremely low temperature were the same. Furthermore, when there were new confirmed cases of COVID-19, the average sentiment score (44) was lower than it (46) when there was no new confirmed case of COVID-19.

**Figure 4:**
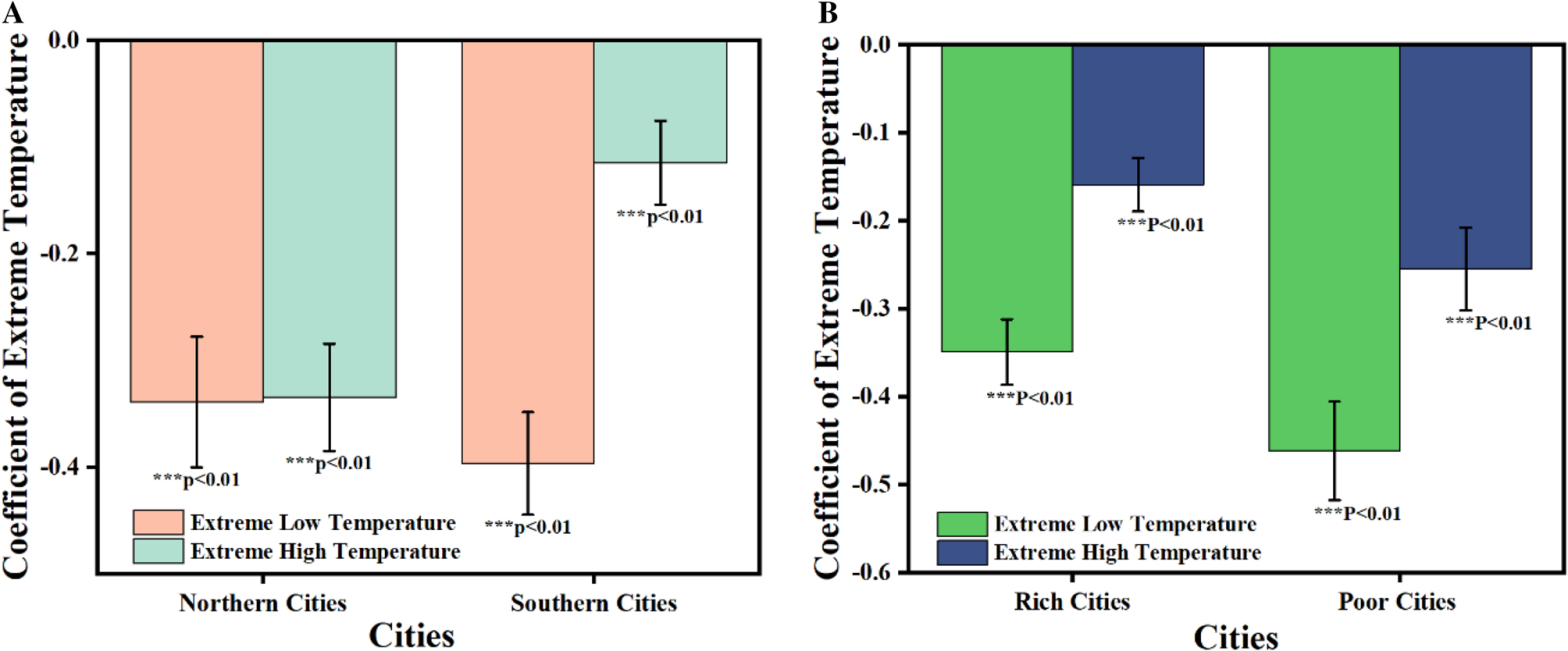
(**A**) The coefficient of extreme temperatures of cities in northern China or southern China. (**B**) The coefficient of extreme temperatures of rich cities or poor cities. Images were drawn using Origin 2018 software by authors.

The latitude and income conditions of cities may also exert influences. We divided our sample cities into two sections based on the Qinling and Huai River line to verify this hypothesis^45^. Our findings indicated that individuals residing in cities with lower latitudes (− 0.1143) were less sensitive to extremely high temperatures compared to those living in cities with higher latitudes (− 0.3345), while residents in areas with higher latitudes (− 0.3389) were less sensitive to extremely low temperatures than those residing in areas with lower latitudes (− 0.3965) (Fig. 4A). Furthermore, we employed the median per capita disposable income of 49 cities as a criterion and categorized them into two groups. Residents in economically disadvantaged cities were significantly more vulnerable to extremely high temperature (− 0.2546) and low temperature (− 0.4616) compared to residents in affluent cities (− 0.1588, − 0.349) (Fig. 4B). Individuals living in wealthier regions in China may have greater access like using air conditioning^46^, to coping mechanisms for dealing with extremely high temperature than those residing in poorer cities do.

## Discussion

This research utilized a near-real-time, multi-step methodology that integrates statistical regression and NLP machine learning techniques to examine the influence of extreme temperatures on residents' sentiment. These approaches assisted us in identifying and measuring the impact of extreme temperatures on the sentiment. In addition, we identified differences in these impacts based on other factors, including the quantity of PM2.5, wind speed, and the presence of COVID-19. The findings provided policymakers with valuable insights into the impact of extreme temperatures on the citizens' sentiment, therefore aiding the formulation of policies to improve public sentiment.

Nevertheless, it is crucial to consider this study's limitations when interpreting these findings. Firstly, the variables in the model were chosen based on an examination of existing research. However, there might be more elements that impact sentiment but were not taken into account. In order to tackle this issue, we incorporated several control variables into the model. By implementing the IV method, we effectively addressed the possible problem of endogeneity that could arise from omitted factors that can influence both extreme temperatures and the sentiment of inhabitants.

Furthermore, it is essential to acknowledge that the sentiment noticed on Weibo may not entirely and precisely reflect the sentiment of all inhabitants. In order to address this constraint, we selected geotagged posts that were published in the Weibo topics of 49 cities. Subsequent iterations of this study would consider incorporating data from additional urban areas in China and multiple social media platforms.

## Conclusion

This research conducted an analysis of 266,744 posts from the 49 cities' topic section of Weibo in China, encompassing 12 months in 2020. Employing multiple regression analysis, this research revealed the substantial correlation between extreme temperatures and residents' sentiment. Subsequently, we utilized variables derived from several sources to substitute the dependent and independent variables in the baseline model to conduct robustness checks. These checks confirmed the adverse influence of extreme temperatures on sentiment. In addition, it was discovered that extremely apparent temperatures and extreme temperature differences negatively affected sentiment. Additionally, this paper discovered that other environmental factors, such as PM2.5 levels and wind, as well as city-specific circumstances like new COVID-19 cases, latitude, and per capita disposable income, may affect the coefficients of extreme temperatures. The variations in the effects of extreme temperatures on sentiment in different settings could provide valuable insights for policymakers.

Moreover, this research offers policymakers a tool to examine the immediate effects of extreme temperatures on residents' sentiment. It could assist them in comprehending the variations in impact across different environmental and urban settings. This study proposes a data-driven and evidence-based approach to implementing policies to improve residents' sentiment. Existing research indicates that negative sentiment may be linked to residents' health and the incidence of crimes^47,48^. Local governments can alleviate extreme temperatures' impact on individuals' sentiment by implementing financial incentives as coping mechanisms when temperatures surpass critical limits. During workdays, when air quality is poor or there are significant adverse public events, the government should employ public resources to reduce the adverse effects of extreme temperatures on residents' sentiment. This can be achieved by implementing measures such as issuing extreme temperature warnings, distributing appropriate medications, and installing extra cooling or heating equipment. These measures will, in turn, help mitigate negative sentiment's adverse impact on public health and criminal activities. The strategies to manage extreme temperatures should be tailored to each city based on its geographical location. Municipal governments in southern China should prioritize the impact of extremely low temperature and proactively implement measures such as issuing early warnings and distributing insulation materials.

Significant strengths lie in our methodology which enables real-time monitoring of changes in residents' sentiment with respect to extreme temperatures by combining various techniques such as MLR, IV approach and NLP technique. Based on this finding, government bodies should consider introducing corresponding policies and appropriate interventions to counteract the negative effects of extreme temperatures and mitigate their impact on residents' sentiment.

### Ethics approval

The data from Weibo posts was publicly available. We used only publicly available data and did not collect data from users with privacy restrictions for our research. We abided by the terms, conditions, and privacy policies of Weibo. We did not seek ethical approval, as all the data were preexisting.

### Supplementary Information

Supplementary Information.

## Data Availability

Empirical analysis data of sentiment, environment and city have been deposited in Github (https://github.com/Baiyixiang/The-effects-of-extreme-temperature-on-public-expressed-sentiment-using-social-media-/). The data of temperatures, precipitation and other environment variables were collected from the National Oceanic and Atmospheric Administration (https://www.noaa.gov/). Figures 1 and 2 were drawn using ArcGIS Pro 3.0.2 by authors^49^. Figures 3 and 4 were drawn using Origin 2018 by authors^50^.
