# Supplementary material for: Effects of extreme temperatures on public sentiment in 49 Chinese cities
Source: Sci Rep. 2024 Apr 30;14:9954. doi: 10.1038/s41598-024-60804-1 (PMC11061318; doi:10.1038/s41598-024-60804-1)
Supplement: Supplementary file 1 — Supplementary Information. [file 41598_2024_60804_MOESM1_ESM.docx]

**Effects of extreme temperatures on public sentiment in 49 Chinese cities**

**Chan Wang**

Professor

School of Economics, Guangdong University of Finance & Economics

Guangzhou, 510320, P. R. China.

Email: **[wangchan0512@163.com](mailto:wangchan0512@163.com)**

**Yi-Xiang Bai**

Postgraduate student, Corresponding author^1^

School of Economics, Guangdong University of Finance & Economics

Guangzhou, 510320, P. R. China

Email: **[terrybai@student.gdufe.edu.cn](mailto:terrybai@student.gdufe.edu.cn)**

**Xin-Wu Li**

School of Economics, Nankai University

Tianjin, 300071, P. R. China.

Email: **[Lixinwu_77@126.com](mailto:Lixinwu_77@126.com)**

**Lu-tong Lin**

Postgraduate student

School of Economics, Guangdong University of Finance & Economics

Guangzhou, 510320, P. R. China

Email: **[Lynn558@163.com](mailto:llutong558@163.com)**

**Supplemental Information**

1. **Data**

**1A. Cities**

These 49 cities analyzed in study are shown in Table S1.

**Table S1. Cities utilized in study.**

| Name | Name | Name | Name | Name | Name | Name |
| --- | --- | --- | --- | --- | --- | --- |
| Baoding | Beijing | Changzhou | Chengdu | Dalian | Dongguan | Foshan |
| Fuzhou | Guangzhou | Guiyang | Harbin | Hangzhou | Hefei | Huizhou |
| Jinan | Jiaxing | Jinhua | Kunming | Lanzhou | Langfang | Nanjing |
| Nanning | Nanchang | Nantong | Ningbo | Qingdao | Quanzhou | Xiamen |
| Suzhou | Shijiazhuang | Shaoxing | Shenzhen | Shenyang | Taizhou | Taiyuan |
| Tianjin | Wenzhou | Wuxi | Wuhan | Xi'an | Xuzhou | Yantai |
| Changchun | Changsha | Zhengzhou | Zhongshan | Chongqing | Zhuhai | Shanghai |

**1B. Data Description**

Table S2 provides information of the major variables’ data sources and definitions. The environment variables are mainly collected from China Statistical Yearbook (CSY), National Oceanic and Atmospheric Administration (NOAA) of the National Environmental Information Center (NCEI) and National Climatic Data Center (NCDC). The COVID-19 diagnose data is collected from Chinese Center for Disease Control and Prevention (CDC).

**Table S2. Major variables’ data sources and definitions.**

| Variable | Name | Role | Resource | Value Range |
| --- | --- | --- | --- | --- |
| 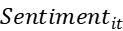 | Expressed Sentiment | Dependent Variable | Tencent NLP | min: 0.27  max: 99.11  mean: 45.90 |
| **** | Robust Expressed Sentiment | Dependent Variable | Baidu  NLP | min: 0.02  max: 99.99  mean: 87.73 |
| 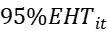 | 95% Extreme High Temperature | Independent Variable in Baseline model | NCEI | min: -23.7  max: 6.64e-06  mean: -4.95 |
|  | Robust 95% Extreme High Temperature | Robust Independent Variable in Baseline mode | Met Office | min: -24.06  max: 4.90  mean: -5.63 |
| 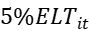 | 5% Extreme Low Temperature | Independent Variable in Baseline model | NCEI | min: -28.22  max: 5.71e-06  mean: -5.03 |
|  | Robust 5% Extreme Low Temperature | Robust Independent Variable in Baseline mode | Met Office | min: -31.34  max: 4.06  mean: -6.44 |
| 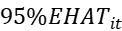 | 95% Extreme High Apparent Temperature | Independent Variable | NCEI | min: -25.92  max: -1.43  mean: -7.23 |
| 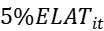 | 5% Extreme Low Apparent Temperature | Independent Variable | NCEI | min: -30.62  max: -1.41  mean: -7.31 |
| 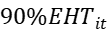 | 90% Extreme High Temperature | Independent Variable | NCEI | min: -19.46  max: 10.01  mean: -3.16 |
| 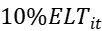 | 10% Extreme Low Temperature | Independent Variable | NCEI | min: -25.93  max: 4.60  mean: -3.56 |
| 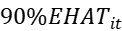 | 90% Extreme High Apparent Temperature | Independent Variable | NCEI | min: -22.06  max: 7.88  mean: -5.44 |
| 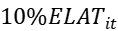 | 10% Extreme Low Apparent Temperature | Independent Variable | NCEI | min: -28.33  max: 2.09  mean: -5.83 |
|  | 90% Extreme High Temperature using 30 years data | Independent Variable | NCEI | min: -26.45  max: 10.01  mean: 10.15 |
|  | 10% Extreme Low Temperature using 30 years data | Independent Variable | NCEI | min: -30.36  max: 3.55  mean: -6.39 |
| 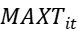 | Daily Maximum Temperature | Temperature Variable | NCEI | min: -30.66  max: 39.45  mean: 22.16 |
| 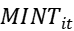 | Daily Minimum Temperature | Temperature Variable | NCEI | min: -28.52  max: 29.66  mean: 12.21 |
| 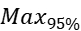 | 95% Standard Daily Maximum Temperature | Threshold Variable | NCEI | min: -6.88  max: 41.03  mean: 27.12 |
| 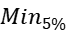 | 5% Standard Daily Minimum Temperature | Threshold Variable | NCEI | min: -38.33  max: 25.11  mean: 7.17 |
| 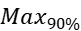 | 90% Standard Daily Maximum Temperature | Threshold Variable | NCEI | min: -8.72  max: 39.57  mean: 25.33 |
| 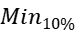 | 10% Standard Daily Minimum Temperature | Threshold Variable | NCEI | min: -33.47  max: 25.96  mean: 8.65 |
| 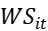 | Daily Wind Speed | Control Variable | NCEI | min: 1.50  max: 20.82  mean: 5.35 |
| 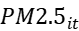 | Daily PM2.5 | Control Variable | NCDC | min: 0  max: 906  mean: 33.75 |
| 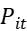 | Daily Precipitation | Control Variable | NCEI | min: 0  max: 157.61  mean: 3.38 |
| 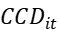 | Daily Confirmed COVID-19 Diagnose | Control Variable | CDC | min: 0  max: 326  mean: 2.53 |
| 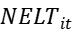 | 5% Neighbor City’s Extreme Low Temperature | Instrumental Variable | NCEI | min: -22.86  max: 4.31e-06  mean: -5.12 |
| 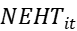 | 95% Neighbor City’s Extreme High Temperature | Instrumental Variable | NCEI | min: -22.87  max: 2.95e-06  mean: -5.11 |
| 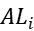 | The Altitude of City i |  | CSY |  |
| 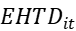 | Extreme High Temperature Difference | Independent Variable | NCEI | min: -24.33  max: 2.95e-06  mean: -5.11 |
| 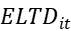 | Extreme Low Temperature Difference | Independent Variable | NCEI | min: -20.62  max: 6.96e-07  mean: -3.10 |
| 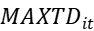 | Daily Maximum Temperature Difference | Temperature Variable | NCEI | min: -18.72  max: 10.28  mean: -0.01 |
| 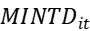 | Daily Minimum Temperature Difference | Temperature Variable | NCEI | min: -12.01  max: 13.14  mean: -0.02 |
| 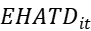 | Extreme High Apparent Temperature Difference | Independent Variable | NCEI | \| min: -26.41  max: -1.53  mean: -8.40 \| \| --- \| |
| 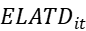 | Extreme Low Apparent Temperature Difference | Independent Variable | NCEI | \| min: -23.44  max: -1.43  mean: -5.38 \| \| --- \| |
| 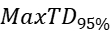 | 95% Standard Daily Maximum Temperature Difference | Threshold Variable | NCEI | min: -1.30  max: 17.46  mean: 4.84 |
| 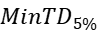 | 5% Standard Daily Minimum Temperature Difference | Threshold Variable | NCEI | min: -15.33  max: 0.12  mean: -3.81 |
| **** | The interaction term between 95% extreme high temperature and daily wind speed | Interaction Variable |  |  |
| **** | The interaction term between 5% extreme low temperature and daily wind speed | Interaction Variable |  |  |
|  | Per Capita Disposable Income in 2022 | Control Variable | National Bureau of Statistics of China | min: 71315  max: 173205  mean: 97178.97 |
|  | The interaction term between 95% extreme high temperature and per capita disposable income | Interaction Variable |  |  |
|  | The interaction term between 5% extreme low temperature and per capita disposable income | Interaction Variable |  |  |
| **** | The interaction term between 90% robust extreme high temperature and daily wind speed | Interaction Variable |  |  |
| **** | The interaction term between 10% robust extreme low temperature and daily wind speed | Interaction Variable |  |  |
|  | The interaction term between 90% robust extreme high temperature and per capita disposable income | Interaction Variable |  |  |
|  | The interaction term between 10% robust extreme low temperature and per capita disposable income | Interaction Variable |  |  |

1. **Control Variables**

**Table S3. Coefficient of correlation of variables**

|  | $\begin{matrix} Expressed \\ Sentiment \end{matrix}$ |  |  |  |  |  |  |  |
| --- | --- | --- | --- | --- | --- | --- | --- | --- |
| $\begin{matrix} Expressed \\ Sentiment \end{matrix}$ | 1.000 |  |  |  |  |  |  |  |
|  | -0.018 | 1.000 |  |  |  |  |  |  |
|  | -0.077 | -0.256 | 1.000 |  |  |  |  |  |
|  | 0.036 | 0.006 | -0.251 | 1.000 |  |  |  |  |
|  | 0.067 | 0.042 | -0.004 | -0.251 | 1.000 |  |  |  |
|  | -0.081 | 0.005 | 0.002 | 0.035 | -0.015 | 1.000 |  |  |
|  | -0.037 | -0.098 | 0.121 | -0.194 | 0.020 | -0.021 | 1.000 |  |
|  | -0.076 | -0.011 | -0.004 | -0.043 | 0.044 | 0.360 | 0.022 | 1.000 |

1. **Instrument Variables**

**3A.**

**Table S4.** **The result of weak IV test (F Statistic)**

| Dependent variables | Expressed Sentiment | Extreme High Temperature | Extreme Low Temperature | Expressed Sentiment |
| --- | --- | --- | --- | --- |
|  |  | Stage 1 of IV | Stage 1 of IV | Stage 2 of IV |
|  | -0.1606^***^ |  |  | -0.1861^***^ |
|  | (0.0273) |  |  | (0.0323) |
|  | -0.2724^***^ |  |  | -0.3766^***^ |
|  | (0.0356) |  |  | (0.0391) |
|  |  | 0.9819^***^  (0.0024) | 0.0005  (0.0018) |  |
|  |  | -0.0022  (0.0029) | 0.9837^***^  (0.0022) |  |
| Constant | 30.7237^***^ | 0.1680 | -0.0013 | 40.2316^***^ |
|  | (0.8032) | (0.0352) | (0.0268) | (0.4574) |
| Control Variables | \| Yes \| Yes \| Yes \| Yes \| \| --- \| --- \| --- \| --- \| | \| Yes \| Yes \| Yes \| Yes \| \| --- \| --- \| --- \| --- \| | \| Yes \| Yes \| Yes \| Yes \| \| --- \| --- \| --- \| --- \| | \| Yes \| Yes \| Yes \| Yes \| \| --- \| --- \| --- \| --- \| |
| City FE  Time FE | Yes | Yes | Yes | Yes |
|  | Yes | Yes | Yes | Yes |
| Solar Terms FE  N | No | No | No | No |
|  | 16944 | 16944 | 16944 | 16944 |
| R^2^ | 0.2822 | 0.9138 | 0.9306 | 0.0205 |
| F Statistic |  | 725.59 | 411.23 |  |

Standard Errors are shown in parentheses, *P<0.1; **P < 0.05; ***P < 0.01.

**3B.**

**Table S5. The result of weak IV test (AR and WALD)**

| Test | Statistic | P-value |
| --- | --- | --- |
| AR | 16.39 | 0.0003 |
| WALD | 26.94 | 0.0000 |

**3C.**

**Table S6. The result of weak IV test (Kleibergen-Paap rk Wald F statistic)**

| Kleibergen-Paap rk Wald F statistic | Stock-Yogo weak ID test critical values |
| --- | --- |
| 4558.239 | 10% maximal IV size: 7.03 |
|  | 15% maximal IV size: 4.58 |
|  | 20% maximal IV size: 3.95 |
|  | 25% maximal IV size: 3.63 |

**3D.**

**Table S7. The result of unidentifiable test**

| Kleibergen-Paap rk LM statistic | Value |
| --- | --- |
| F | 45.179 |
| P-val | 0.0000 |

The F statistics are both greater than 10 proving that the two instrumental variables pass the weak IV test. Moreover, the p-value of AR and WALD are both less than 0.01 proving that two instrumental variables pass the weak IV test. Furthermore, the Kleibergen-Paap rk Wald F statistic is greater than the 10% maximal IV size of Stock-Yogo weak ID test critical values, and the p-value of Kleibergen-Paap rk LM statistic is less than 0.01 proving two instrumental variables pass the weak IV and unidentifiable test.
